# Zygomaticomaxillary complex fractures: finding the least complicated surgical approach (A Randomized Clinical Trial)

**DOI:** 10.1186/s12903-023-03249-8

**Published:** 2023-08-04

**Authors:** Lydia N. Melek, Marwa G. Noureldin

**Affiliations:** Department of Oral and Maxillofacial Surgery, Faculty of Dentistry, Alexandria University, Champolion Street, Azarita, Alexandria Egypt

**Keywords:** Zygomaticomaxillary, Fractures, Surgical approach, Complications

## Abstract

**Background:**

Many approaches have been suggested for management of zygomaticomaxillary fractures. Each approach has its own advantages and limitations.

**Aim of this study:**

The study is intended to compare between the subtarsal approach, conventional transconjuctival approach and the Y- modification of the transconjuctival approach in the management of zygomatico-maxillay complex fractures.

**Materials and methods:**

Twenty-four patients with age range of 20–50 years requiring open reduction and fixation of a fractured zygomatic complex were randomly divided into three equal groups. Group A: subtarsal approach group, group B: a conventional transconjunctival approach group and group C: Y- modification of the transconjunctival approach group. Intraoperative and postoperative parameters were evaluated.

**Results:**

As for the exposure time, group C had the longest duration. Easy access to the site of fracture was reached in all groups with no statistically significant difference. During the first 24 h, the pain was only statistically significant between groups A and B with higher pain level in group A. After the first week, pain was significantly higher in groups A and C, with respect to group B. The least edema was observed in group B after 24 h, one week and four weeks postoperatively. Regarding ocular complications, wound healing and sensory nerve function, there was no statistically significant difference between the groups. Scarring was only noticeable in group A patients.

**Conclusion:**

The transconjunctival approach provides adequate exposure with excellent esthetics and minor complications. The Y-modification also delivers an esthetic access with inconspicuous scar to the frontozygomatic region.

**Trial registration:**

The trial has been registered on clinicaltrials.gov (ID: NCT05695872).

## Introduction

The zygomaticomaxillary complex (ZMC) is a tetrapod structure that involves articulation with the maxilla, frontal, temporal, and sphenoid bones [1]. It is commonly involved in maxillofacial fractures with a percentage ranging between 23 and 42% of such fractures according to previous studies [2, 3].

Being a major buttress of the face, the ZMC integrity is crucial to function and aesthetics of the facial skeleton. To restore the fractured ZMC to the proper anatomical position, adequate exposure of the fracture site is of high importance [4]. Improper ZMC reduction may lead to facial asymmetry, altered sensation of the infraorbital nerve, affection of ocular function and/or restriction of normal mandibular movement [1].

More than 70% of ZMC fractures are managed surgically using open reduction and internal fixation, where access to the inferior orbital rim is obtained through either transcutaneous or transconjunctival approach. Transcutaneous approaches include the infraorbital, subtarsal and subciliary incisions which provide good accessibility to the fracture site but on the expense of the associated scar formation [5, 6]. The subciliary approach is done about 2 mm, while the subtarsal approach is done in a natural crease usually 5 to 7 mm below the ciliary margin, respectively [7].

The transconjunctival approach has been advocated for avoidance of the undesirable scar in an exposed region of the face. Another important advantage of the transconjunctival approach is that it provides visualization of the orbital floor and inferior orbital rim without interference of the lacrimal drainage system [8]. This approach which is performed through the conjunctiva below the level of the tarsus followed by preseptal or retroseptal dissection to the orbital rim, has also been proved to be a convenient and less time-consuming approach [1].

However, some limitations were reported in terms of accessibility to reduce the fracture and apply plates for fixation. In addition, technical skills, proper eye protection and gentle retraction of tissues are required to prevent complications [9].

Several modifications for the original transconjunctival approach have been suggested to overcome the limitations, provide wider access, and still benefit from the hidden incision advantage. One of the modifications was the Y-modification of the cutaneous portion of the transconjunctival approach with lateral canthotomy proposed by Martinez and Bradrick [10].

Over the previous decades, several approaches were used for management of the orbital region of zygomaticomaxillary fractures. However, no single approach has yet been proved to be the best option concerning maximum accessibility and least complication rate.

The present study was performed to compare three surgical approaches to ZMC fractures: the subtarsal approach, the conventional transconjunctival approach (with or without lateral canthotomy) and the transconjunctival approach with Y- modification.

## Patients and methods

### Study design

The study is a randomized clinical trial, following the Consolidated Standards of Reporting Trials (CONSORT) guidelines [11]. The trial has been registered on clinicaltrials.gov (ID: NCT05695872) and trial registration posted on 25/01/2023. The research protocol was approved by the Research Ethics Committee of Alexandria University Faculty of Dentistry (IRB No. 001056 – IORG 0008839).

### Study sample

Sample size was estimated assuming 80% study power and 5% alpha error. El-Anwar et al. [9] reported mean (SD) duration from incisions to fracture exposure = 13.7 (2.17) minutes in the subciliary approach, and 14.6 (2.31) minutes in the transconjunctival approach. Yassin et al. [12] reported mean (SD) exposure duration = 20.00 (3.41) minutes in Y-modification of the transconjunctival approach. Based on comparison of means, using two-tailed test, the minimum sample size was calculated to be 7 patients per group, increased to 8 to make up for cases lost to follow-up. The total required sample size = number of groups × number per group = 3 × 8 = 24 patients [13].

Software

G*Power 3.1.9.4

### Study setting and location

Participants were selected from the Emergency Ward of Alexandria university teaching hospital from August 2022 to October 2022 and were operated under the authority of the oral and maxillofacial surgery department, faculty of dentistry, Alexandria University. Patients were informed about the procedure details and each patient signed an informed consent.

### Criteria for patient selection

#### Inclusion criteria

- Patients with ZMC fractures requiring open reduction and internal fixation.
- Adult patients aged between 20 and 50 years with no gender predilection.

#### Exclusion criteria

- An existing laceration in the inferior and lateral periorbital site.
- Infection at the fracture line.
- Comminuted fracture with bone loss.
- Acute and chronic conjunctival diseases.

### Randomization

The enrolled patients (*n* = 24) were randomly allocated by the authors (LM and MN) into three equal groups involving simple randomization method [14] using a computerized random number generator software [15]. The numbers were hidden in sealed envelopes with an allocation ratio of 1:1:1.

### Grouping of the patients

#### Group A: (*n* = 8)

Eight patients were treated using the subtarsal approach.

#### Group B: (*n* = 8)

Eight patients were treated with conventional transconjunctival approach.

#### Group C: (*n* = 8)

Eight patients were treated with transconjunctival approach with Y modification.

## Methods

### Preoperative assessment

#### History

Full detailed personal data, past medical and dental history and the chief complaint were registered including including cause, time, date, place and type of assault.

#### General examination

Extra-oral and intaoral examination were performed for all patients through inspection and palpation.

#### Radiographic examination

Computed tomography scans were obtained for all patients preoperatively.

### Surgical procedure

- Preoperative patient preparation

Patients underwent the essential laboratory investigations for operation clearance. They were instructed to fast for 8 h before surgery.

Cefotaxime (Cefotax: each vial contains cefotaxime (as sodium salt) 1 gm, manufactured by E.I.P.I.C.O.)1gm/12 intravenously were given preoperatively as prophylaxis to prevent postoperative infection.

b) Operative procedure

1. The patients were operated under general anaesthesia.
2. The surgical field was disinfected with povidone-iodine solution, then drapped with sterile towels showing the site of surgery only.

#### Group A [1]

**Fig. 1:**
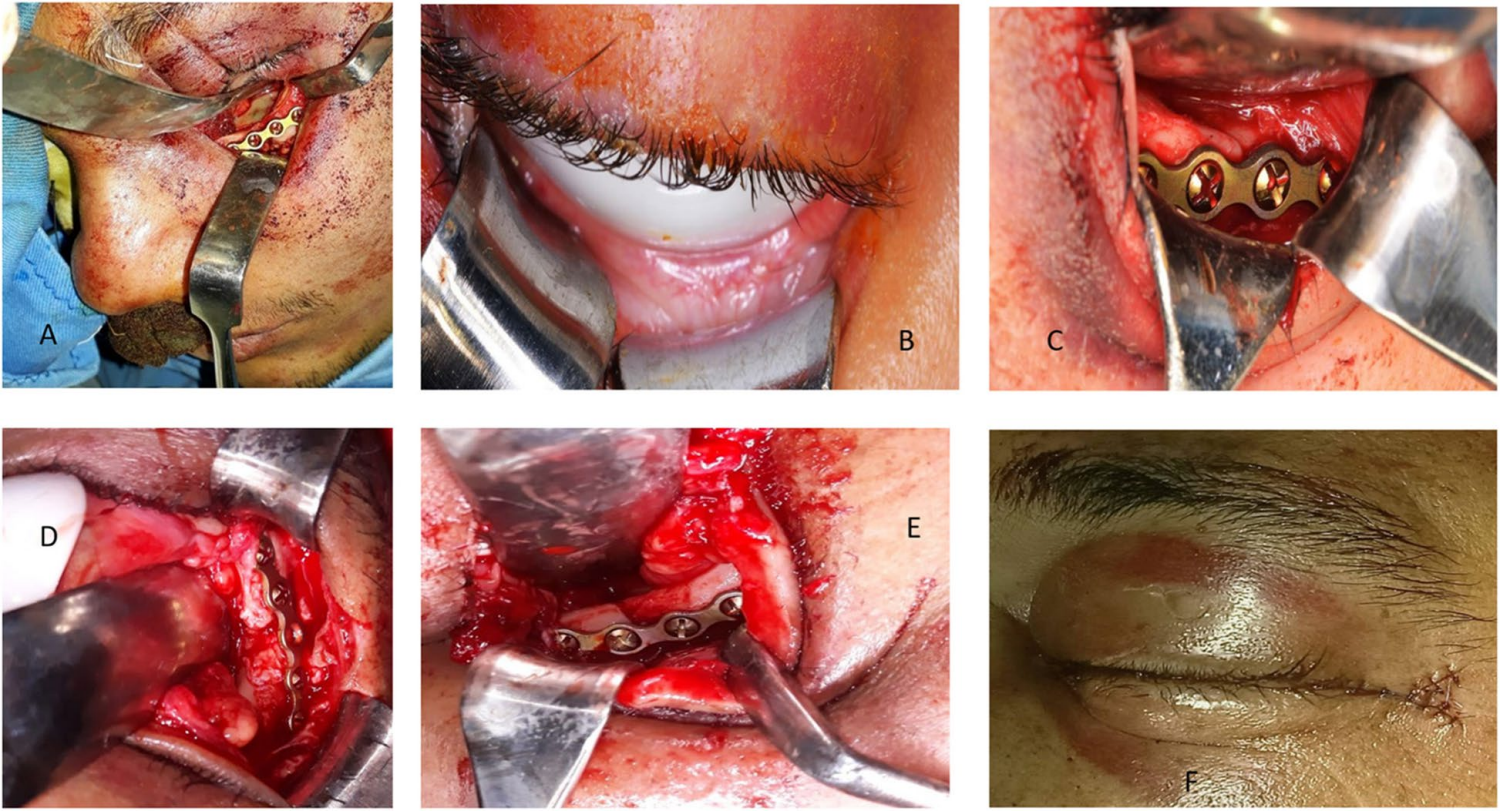
**A** Miniplate fracture fixation at infraorbital rim through subtarsal approach. **B** Corneal shield placement. **C** Miniplate fracture fixation at infraorbital rim through conventional transconjuctival approach. **D** Miniplate fracture fixation at frontozygomatic suture through Y modification transconjuctival approach. **E** Fixation at infraorbital rim through Y modification transconjuctival approach. **F** Closure of the Skin Y-shaped incision

1. A temporary tarsorrhaphy was performed then removed at the end of the operation.
2. A vasoconstrictor (Adrenaline 1/200,000 concentration) was infiltrated through the skin only then, a subtarsal incision was made approximately 4–5 mm below the lashes, along the whole lid.
3. Subcutaneous dissection was proceeded towards the orbital rim using sharp dissection with a scalpel or scissors.
4. A hemostat was used to dissect through the orbicularis oculi muscle deep to the periosteum of the lateral orbital rim.
5. A scalpel incision through the orbicularis oculi muscle then the periosteum on the anterior maxillary and zygomatic surfaces, just 2 to 3 mm inferior to the orbital rim.
6. A periosteal elevator was used to strip the periosteum and expose the bone surface.
7. Reduction was performed and fixation of the fracture was secured using miniplates (Fig. 1A).
8. Closure was executed in two layers starting with the periosteum then the skin through vicryl resorbable sutures for the former and non-resorbable suture for the latter.
9. The eye was cleaned by copious rinses with saline solution and a lower eyelid suspensory suture (frost suture) was secured and taped to the forehead to lessen the occurrence for vertical lower eyelid shortening during healing. It was then removed after 1 week.

#### Group B [1, 16]

1. The globe was protected using a corneal shield (Fig. 1B)
2. A vasoconstrictor was injected beyond the conjunctiva to help with hemostasis.
3. The lower eyelid was everted delicately with a forceps and traction sutures which were passed from palpebral conjunctiva to skin, 4 to 5 mm inferior to the lid margin.
4. If needed, lateral canthotomy was done, if fixation at frontozygomatic suture was required.
5. Then, a hemostat dissected the conjunctiva without extending medially farther than the lacrimal punctum.
6. The incision through the periorbital tissue was performed in a retroseptal manner. A broad retractor was adjusted posteriorly to the infraorbital rim, restricting the orbital fat.
7. A periosteal elevator was used to cut the periosteum over the orbital rim and maxillary anterior surface, the zygoma, and the floor of the orbit.
8. Reduction was performed and fixation of the fracture was secured by miniplates (Fig. 1C).
9. The conjunctiva was sutured with 6–0 vicryl.
10. The eye was cleaned with saline solution and a frost suture was applied for a period of 1 week.

#### Group C [12]

1. A corneal shield was placed.
2. A Y-shaped line was marked on the lateral aspect of the lateral canthal angle at the skin crease.
3. Local anaesthesia vasoconstrictor was injected subcutanoeusly below the marked line at the zygomatic area across the infraorbital rim.
4. Traction sutures were performed for tarsorrhaphy, or a lower eyelid retractor was used.
5. The skin incision was made in a Y-shaped manner and the lateral canthal was cut to separate the tarsal plates.
6. With one arm of the scissor placed inside the tunnel and the other arm on the outside of the conjunctiva, sectioning of the conjunctiva was executed with caution to stay 5 mm below the lower tarsal plate.
7. After the canthotomy, the conjunctiva was exposed and a tunnel beneath the conjunctiva was created using blunt hemostat all the way toward the medial end of the incision.
8. Transconjunctival incision and dissection were performed in the same manner as in group B to expose the fracture at the frontozygomatic region.
9. The cutaneous Y incision transformed into a box when retracting its corners, widening field exposure and allowing better access to the fronto-zygomatic region, lateral orbital wall, and the orbital floor with one approach.
10. Reduction and fixation of the fracture was accomplished using miniplates (Fig. 1D&E).
11. The superficial portion of the lateral canthal ligament was sutured to the temporal aponeurosis using 3–0 vicryl. The conjunctiva was closed with 6–0 vicryl and the skin over the lateral canthus is sutured in the original Y shape with 6–0 prolene (Fig. 1F).
12. The eye was cleaned and a frost suture was applied for 1 week.

### Post-operative phase

- Early postoperative care

All patients were advised on application of ice pack extra-orally immediately postoperatively for 12 h.

b) Postoperative medication

- Intravenous cefotaxime 1 gm/12 h on the first day then amoxicillin + clavulanate (Augmentin: amoxicillin 875mg + clavulanic acid 125 mg: GlaxoSmithkline, UK) 1 gm twice daily for 5 days.
- Metronidazole (Flagyl: metronidazole 500mg by GlaxoSmithkline, UK) 500 mg every8 hours for 5 days.
- Alpha-chemo-trypsin (Alpha-chemo-trypsin: Leurquin France, packed by Amoun pharmaceutical CO.S.A.E-Egypt)ampules once daily for 5 days.
- Diclofenace potassium (Cataflam: diclofenac potassium 50 mg: Novartis-Switzerland) 50 mg for 5 days.
- Patients were advised to use antiseptic chlorhexidine (Hexitol: chlorhexine125mg/100 ml, concentration0.125%: Arabic drug company, ADCO) mouth wash in situations where additional vestibular maxillary intraoral incision was performed.
- A high protein, soft, high calorie diet was instructed for all patients postoperatively.

### Parameters for evaluation

Follow-up was done regularly for all patients till 3 months postoperative, and the following criteria were assessed:

1. Exposure duration

The duration from performing the incision till the field exposure calculated in minutes.

2. Accessibility to fracture site

Adequate exposure of the infraorbital rim, orbital floor and medial orbital wall provided by the incision and accessibility to proper fracture reduction and fixation.

3. Postoperative pain [17]

Pain was evaluated at 24 h and at 1 week according to a 10-point Visual Analogue Scale (VAS), (0–1 = none,2–4 = mild,5–7 = moderate,8–10 = severe).

4. Postoperative edema

Postoperative edema was subjectively evaluated at 24 h, 1 week and 4 weeks to be assessed into mild, mild to moderate, moderate to severe and severe.

5. Postoperative ocular complications

Complications such as ectropion, entropion, corneal abrasion, scleral show, enophthalalmous, or impaired movement of the eye.

6. Wound healing

The sutured wounds were examined for presence of infection or any disturbance in wound healing as hardware exposure and/or wound dehiscence.

7. Sensory nerve function

Subjective assessment of the infraorbital nerve sensation by patient questioning about any alteration in sensation at 3 months postoperatively.

Objective assessment by dental probe pressure to assess sensory changes along the distribution of the infraorbital nerve with contralateral side comparison (nociceptive method) [1, 12].

8. Postoperative scar and esthetic appearance

Postoperative scarring was recorded as noticeable or unnoticeable at 6 weeks postoperatively and as a result, esthetic affection was evaluated.

### Radiographic evaluation

Immediate postoperative computed tomography was requested for adequacy of fracture reduction assessment.

### Statistical analysis [18, 19]

With the aid of the IBM SPSS software package version 20.0, data were fed into the computer and evaluated (Armonk, NY: IBM Corp). Number and percentage were used to describe qualitative data. The normality of the distribution was examined using the Shapiro–Wilk test. The range (minimum and maximum), mean, standard deviation, and median were used to characterise quantitative data. At the 5% level, significance of the results was determined. The tests used were:

1. The Chi-square test compares two sets of categorical variables.
2. Monte Carlo correction: was used as correction for chi-square in case of > 20% of the cells having expected count < 5
3. F-test (One-Way ANOVA): For normally distributed numerical variables, to compare across more than two groups, and Post Hoc test (Tukey) for pairwise comparisons.
4. Paired t-test: Used to compare two periods of normally distributed quantitative data.

## Results

Twenty-four patients, 19 males (79.2%) and 5 females (20.8%) with age ranging from 20 to 50 years old with a mean of 31.82 ± 9.23 years were enrolled in the study (Table 1). The patients were selected from the Emergency Ward of Alexandria university teaching hospital and were operated under the authority of the oral and maxillofacial surgery department, faculty of dentistry, Alexandria University.

**Table 1:** Demographic data in each group

Demographic data/Group	Group A (*n* = 8)	Group B (*n* = 8)	Group C (*n* = 8)
Age: Min- max	22–50 years	21–43 years	20–42 years
Gender	5 Males + 3 Females	6 Males + 2 Females	7 Males + 1 Female
Side affected	5 Left + 3 Right	4 Left + 4 Right	6 Left + 2 Right

**Fig. 2:**
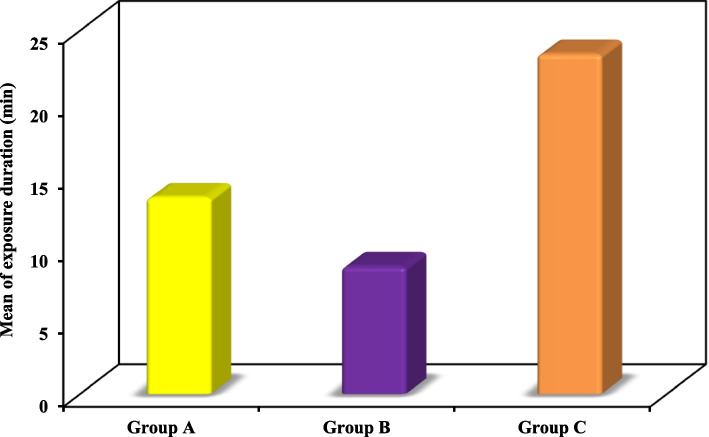
Comparison between the three studied groups according to exposure duration

1. Exposure duration: Fig. 2, Table 2

**Table 2:** Comparison between the three studied groups according to accessibility and exposure time

	**Group A** **(** ***n*** ** = 8)**		**Group B** **(** ***n*** ** = 8)**		**Group C** **(** ***n*** ** = 8)**		**Test of Sig**	**P**
	**No**	**%**	**No**	**%**	**No**	**%**		
**Accessibility to fracture site**	8	100	8	100	8	100	–	–
**Exposure duration (min)**								
Mean ± SD	13.50 ± 1.77		8.75 ± 1.28		23.38 ± 1.41		F = 197.39^*^	< 0.001^*^
Median (Min. – Max.)	13.50 (11.0 – 16.0)		8.50 (7.0 – 11.0)		23.50 (21.0 – 25.0)			
**Sig. bet. grps**	p_1_ < 0.001^*^,p_2_ < 0.001^*^,p_3_ < 0.001^*^							

*SD* Standard deviation, *F* F for One way ANOVA test, Pairwise comparison bet. each 2 groups was done using Post Hoc Test (Tukey)

p: *p* value for comparing between the three studied groups

p_1_: *p* value for comparing between Group A and Group B

p_2_: *p* value for comparing between Group A and Group C

p_3_: *p* value for comparing between Group B and Group C

^*^: Statistically significant at *p* ≤ 0.05

As for the exposure time measured from performing the incision till the field exposure, group C had the longest duration, followed by group A then group B having the shortest duration. The values had statistically significant difference between all groups.

2. Accessibility to fracture site: Table 2

Regarding the accessibility to the site of fracture, easy access was reached in all groups with no statistically significant difference.

3. Postoperative pain

Regarding the pain during the first 24 h, the pain was only statistically significant between groups A and B with higher pain level in group A. After the first week, pain was statistically significant higher in groups, A and C, with respect to group B. On the other hand, the difference between both groups A and C was not statistically significant Table 3.

**Table 3:** Comparison between the three studied groups according to postoperative pain (visual analogue scale)

Pain	Group A (*n* = 8)	Group B (*n* = 8)	Group C (*n* = 8)	F	P
**24 h**					
Mean ± SD	9.0 ± 0.76	7.75 ± 1.04	8.38 ± 0.92	3.777^*^	0.040^*^
Median (Min. – Max.)	9.0 (8.0 – 10.0)	8.0 (6.0 – 9.0)	8.0 (7.0 – 10.0)		
**Sig. bet. grps**	p_1_ = 0.031^*^,p_2_ = 0.372,p_3_ = 0.372				
**1 week**					
Mean ± SD	6.63 ± 0.92	4.0 ± 0.76	5.75 ± 1.39	12.840^*^	< 0.001^*^
Median (Min. – Max.)	7.0 (5.0 – 8.0)	4.0 (3.0 – 5.0)	6.0 (4.0 – 7.0)		
**Sig. bet. grps**	p_1_ < 0.001^*^,p_2_ = 0.244,p_3_ = 0.009^*^				

*SD* Standard deviation, *F* F for One way ANOVA test, Pairwise comparison bet. each 2 groups was done using Post Hoc Test (Tukey)

p: *p* value for comparing between the three studied groups

p_1_: *p* value for comparing between Group A and Group B

p_2_: *p* value for comparing between Group A and Group C

p_3_: *p* value for comparing between Group B and Group C

^*^: Statistically significant at *p* ≤ 0.05

**Fig. 3:**
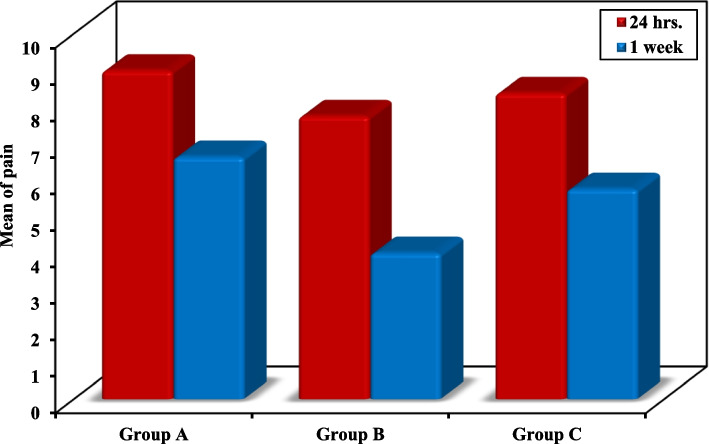
Comparison between 24 h. and 1 week according to postoperative pain (visual analogue scale)

When comparing pain in each group, pain after 1 week was statistically significantly lower than after 24 h in all groups Fig. 3.

**Fig. 4:**
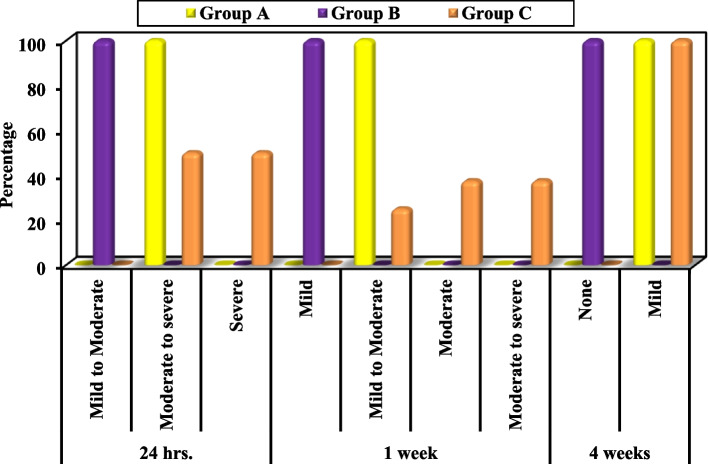
Comparison between the three studied groups according to postoperative edema

4. Postoperative edema: Fig. 4, Table 4

**Table 4:** Comparison between the three studied groups according to postoperative edema

Edema	Group A (*n* = 8)		Group B (*n* = 8)		Group C (*n* = 8)		χ^2^	P
	**No**	**%**	**No**	**%**	**No**	**%**		
**24 h**								
Mild to Moderate	0	0.0	8	100	0	0.0	27.228^*^	^MC^p < 0.001^*^
Moderate to severe	8	100	0	0.0	4	50.0		
Severe	0	0.0	0	0.0	4	50.0		
**Sig. bet. grps**	^FE^p_1_ < 0.001^*^,^FE^p_2_ = 0.077,^MC^p_3_ < 0.001^*^							
**1 week**								
Mild	0	0.0	8	100	0	0.0	30.386^*^	^MC^p < 0.001^*^
Mild to Moderate	8	100	0	0.0	2	25.0		
Moderate	0	0.0	0	0.0	3	37.5		
Moderate to severe	0	0.0	0	0.0	3	37.5		
**Sig. bet. grps**	^FE^p_1_ < 0.001^*^,^MC^p_2_ = 0.008^*^, ^MC^p_3_ < 0.001^*^							
**4 weeks**								
None	0	0.0	8	100	0	0.0	23.289^*^	^MC^p < 0.001^*^
Mild	8	100	0	0.0	8	100		
**Sig. bet. grps**	^FE^p_1_ < 0.001^*^,p_2_ = -, ^FE^p_3_ < 0.001^*^							

χ^2^ Chi square test, *FE* Fisher Exact, *MC* Monte Carlo

p: *p* value for comparing between the three studied groups

p_1_: *p* value for comparing between group A and group B

p_2_: *p* value for comparing between group A and group C

p_3_: *p* value for comparing between group B and group C

^*^: Statistically significant at *p* ≤ 0.05

The least edema was observed in group B after 24 h, one week and four weeks postoperatively compared to groups A and C. These values were statistically significant. When comparing group C to Group A, the edema was statistically significantly lesser in Group C only at one week post operative.

**Fig. 5:**
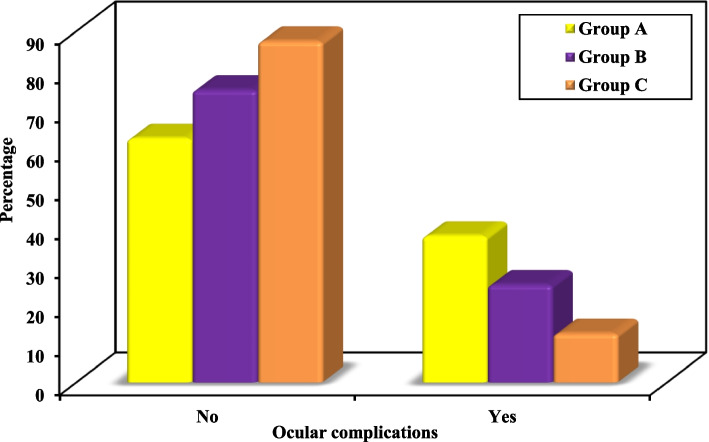
Comparison between the three studied groups according to Ocular complications

**Fig. 6:**
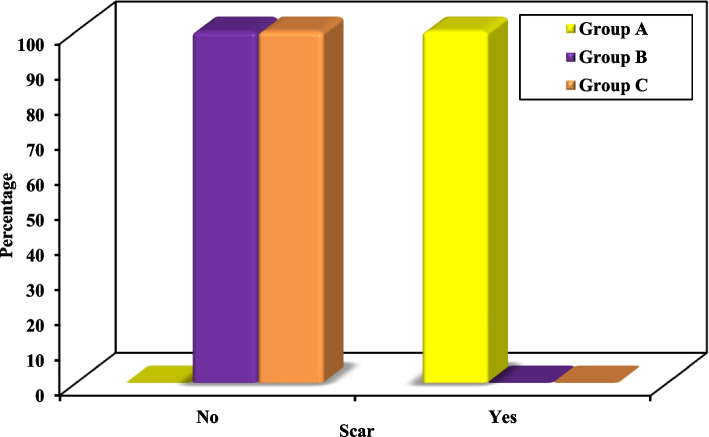
Comparison between the three studied groups according to scar

5. Postoperative ocular complications, wound healing, sensory nerve function, scar and esthetic appearance: Figs. 5 and 6, Table 5

**Table 5:** Comparison between the three studied groups according to ocular complications, wound healing, sensory nerve function and scar

	**Group A** **(** ***n*** ** = 8)**		**Group B** **(** ***n*** ** = 8)**		**Group C** **(** ***n*** ** = 8)**		**χ** ^**2**^	**P**
	**No**	**%**	**No**	**%**	**No**	**%**		
**Ocular complications**								
No	5	62.5	6	75.0	7	87.5	1.358	^MC^p = 0.836
Yes	3	37.5	2	25.0	1	12.5		
**Sig. bet. grps**	^FE^p_1_ = 1.0,^FE^p_2_ = 0.569,^FE^p_3_ = 1.0							
**Wound healing**								
Normal	6	75.0	8	100	7	87.5	4.721	^MC^p = 0.302
Distrubed (Infection)	2	25.0	0	0.0	0	0.0		
Wound dehiscence	0	0.0	0	0.0	1	12.5		
**Sig. bet. grps**	^FE^p_1_ = 0.467,^MC^p_2_ = 0.469,^FE^p_3_ = 1.0							
**Sensory nerve function**								
Not affected	6	75.0	8	100	7	87.5	2.092	^MC^p = 0.747
Affected (Numbnes)	2	25.0	0	0.0	1	12.5		
**Sig. bet. grps**	^FE^p_1_ = 0.467,^FE^p_2_ = 1.000,^FE^p_3_ = 1.0							
**Scar**								
No	0	0.0	8	100	8	100	23.289^*^	^MC^p < 0.001^*^
Yes	8	100	0	0.0	0	0.0		
**Sig. bet. grps**	^FE^p_1_ < 0.001^*^,^FE^p_2_ < 0.001^*^,p_3_ = _							

χ^2^ Chi square test, *FE* Fisher Exact, *MC* Monte Carlo

p: *p* value for comparing between the three studied groups

p_1_: *p* value for comparing between group A and group B

p_2_: *p* value for comparing between group A and group C

p_3_: *p* value for comparing between group B and group C

^*^: Statistically significant at *p* ≤ 0.05

The ocular complications in group A included only 3 out of 8 patients with a complaint of ectropion and scleral show. In group B, only 1 patient showed slight entropion which had no negative effect on the integrity of the cornea nor the conjunctiva. Another patient in group B showed transient diplopia which resolved by the end of the 2^nd^ post operative week. In group C, only 1 patient had mild scleral show which was acceptable by the patient.

Regarding ocular complications, wound healing and sensory nerve function, there was no statistically significant difference between any of the groups.

With respect to scarring, statistically significant difference was only found in groups B and C when compared to group A; groups B and C, showed no scar in all of the cases while Group A showed scars in all of the cases.

6. Radiographically, all cases of the 3 groups showed adequate reduction and fixation of the fracture when compared to the contralateral side

## Discussion

Several incisions have been used by maxillofacial surgeons to approach the orbital rim in ZMC fractures and have been investigated to reach the most accessible approach with the least complication rate. The aim of our study was comparing three surgical approaches: the subtarsal approach, the conventional transconjunctival approach and the transconjunctival approach with Y modification.

Twenty-four patients were included in our study; 79.2% of them were males and 20.8% were females with a ratio of 3.8:1. This agrees with several previous studies showing male predominance in maxillofacial trauma [3, 20].

Our results have shown a significant difference concerning the operative time starting from the incision till exposure of the fracture site. The group of transconjunctival approach with Y modification had the longest duration (23.38 ± 1.41 min), followed by the subtarsal approach group (13.50 ± 1.77 min) then the conventional transconjunctival approach group having the shortest duration (8.75 ± 1.28 min). This disagrees with Novelli et al. who assumed that both the transconjunctival and palpebral approaches take nearly the same operative time [8]. Also a non-significant difference was detected by El-Anwar et al. between the subciliary and transconjunctival approaches in terms of mean duration from incision to fracture exposure (13.7 ± 2.17 min in subciliary approach and 14.6 ± 2.31 min in transconjunctival approach with lateral canthotomy) [9]. A longer mean exposure duration was recorded by Santosh and Giraddi for the transconjunctival approach (21 min) [21].

Exposure duration with comparable results to the Y cutaneous modification approach is the transconjuctival approach with lateral canthotomy. Subramanian et al. recorded a familiar duration (22 min) as well as Holtmann et al. (20 min) when performing the transconjuctival approach with lateral canthotomy [5, 22].

Differences in exposure time between studies may be partially related to the learning curve, expertise and technical skills of the operator managing each surgical approach. Specifically, the longer duration of the transconjunctival approach with Y modification may be attributed to the extended cutaneous Y incision that necessitated more time for the added tissue dissection. Moreover, the transconjuctival approach necessitates the meticulous reconstruction of the lateral canthal tendon to avoid mal-positioning of the lid caused by any canthal asymmetry [10, 23].

Concerning accessibility to the surgical site, easy access to infraorbital rim, orbital floor and medial orbital wall was achieved in all groups of our study with no statistically significant difference. This is in accordance with Novelli et al. who have proved through their research on 56 cases that the transconjunctival approach provides very good visualization to the orbital floor and does not interfere with the lacrimal drainage system and they claimed that it is the best surgical approach in terms of function and esthetics. Moreover, they revealed the absence of any long term complications [8]. On the other hand, El-Anwar et al. have found that the transconjunctival approach alone was not sufficient for proper exposure and that lateral canthotomy was needed due to the limited surgical field [9].

In our study, for all of the 8 cases in group C, the cutaneous Y-modification offered the surgeon a profound accessibility to both; the inferior and lateral orbital rims omitting the necessity for a supplementary lateral eyebrow incision. The resultant box shaped window created by the retraction of Y incision of the transconjunctival approach provided a wide and easy visibility to both fracture sites. This coincided with Ilankovan et al. who conducted the Y modification technique declaring that simultaneous exposure to both the inferior orbital and the lateral orbital rims was the most practical compared to other approaches [24].

Earlier reports on the Y modifications come in agreement with the findings of our study, where sufficient exposure was obtained Martinez et al. (2012), in the 24 subjects, and by Rajkumar et al. (2016), in 10 cases [10, 23]. The Y-modification of the transconjunctival approach provides a good exposure of the surgical field, taking into consideration complete knowledge of the anatomical details of the lateral canthus region, as suggested by Rajkumar et al. [23].

After the first week, pain was significantly higher in groups, A (subtarsal approach) and C (transconjunctival approach with Y modification), with respect to group B (conventional transconjunctival approach). This contrasts with the findings of El-Anwar et al. who have reported a non-significant difference in the mean pain score between the subciliary and transconjunctival approaches [9].

For the 3 groups in this study, there was a statistically significant decrease in pain after 1 week compared to 24 h postoperatively. This was in accordance with Dickinson and Gausas and another research by Shoukath et al. in which pain regression was linked to the decline in the value of the postoperative edema [16, 25].

The least postoperative edema was observed in group B after 24 h, one week and four weeks compared to groups A and C. These values were statistically significant. Nevertheless, El-Anwar et al. have found periorbital edema in all patients of the subciliary and transconjunctival approaches during the first postoperative week being more severe in the transconjunctival group (with lateral canthotomy) [9]. Periorbital edema in the early postoperative period is related to the damage of the lacrimal drainage system induced by incision and dissection at the lateral canthus area [16].

With respect to scarring, groups B and C showed no scar in all cases while Group A showed scars in all cases. The Y modification of the transconjunctival approach yielded the most inconspicuous and well-masked scar, since having the advantage of being hidden in the crow’s feet skin crease which is a natural feature. Our findings are consistent with those of Rajkumar et al. where a good cosmetic result was obtained through the Y modification of the transconjunctival approach due to hiding the small cutaneous scar within a natural skin crease [23]. Similarly, Santosh and Giraddi have described the transconjunctival approach as the most esthetic approach to the infraorbital rim, orbital floor and medial wall of the orbit [21]. Conversely, a study by Oztel et al. has shown that the subtarsal approach resulted in non-visible scar formation in 61 to 76.5% of patients, while providing a straightforward, and accessible approach to the orbital rim [26].

Regarding ocular complications, wound healing and sensory nerve function, there was no statistically significant difference between any of our study groups. This goes hand in hand with the study by Oztel et al. where the difference in overall complication rate was not statistically significant between the subtarsal approach group and the transconjunctival approach group, however; their need for reoperation was higher in the transconjunctival group [26].

Only two cases of the subtarsal group and one case of the Y-modification group of the present study reported affection of the infraorbital sensory nerve function. However, all patients have completely recovered and regained normal sensation by the end of the follow up period. A similar finding was reported by Yassin et al. [12].

Our study has shown ocular complications in 37.5% of cases with subtarsal approach, in 25% of cases with conventional transconjunctival approach and in 12.5% of cases with modified transconjunctival approach. All the ocular complications were minimal and transient, causing no significant disturbance to the patients. Using a corneal shield for globe protection throughout the operation and application of a suspensory frost suture at the end of the surgical procedure helped greatly in minimizing postoperative ocular complications. Ridgway et al. performed transconjunctival approach and recorded entropion in 2 of the cases along a period of 10 years and contributed these results to the lack of exposure of the field that compelled extensive retraction [27].

Wray et al. have reported an incidence of ectropion in 42% of cases with subciliary approach in comparison to no ectropion in patients with transconjunctival approach [28]. Minor scleral show has been found by Rajkumar et al. in two out of ten patients with Y-modification of the transconjunctival approach but without clinical significance [23]. In another study, ectropion was detected in 10% and scleral show in 15% of the subciliary approach group while transient entropion was found in 20% of the transconjunctival approach group [9]. Devi et al. when compared the transconjunctival Y modification approach to the subtarsal with the lateral brow approach, recorded no ectropion with the transconjunctival Y modification to 7.7% occurrence with the subtarsal approach [29].

However, the present study had few limitations including the inability to apply blinding to the patients or operators due to the obvious nature of the incision, in addition to the relatively short follow up period.

## Conclusion

Within the limitations of the current study, we can conclude that the transconjunctival approach (whether conventional or Y-modified) provides adequate exposure to the infraorbital rim, orbital floor and medial orbital wall with excellent esthetics and minor complications. The Y-modification delivers an esthetic access with inconspicuous scar to the frontozygomatic region which obviates the need for an additional lateral eyebrow incision. Moreover, it provides a wide surgical field in the periorbital area, but with longer exposure duration.

## Data Availability

The datasets generated and /or analysed during the current study are publicly available from the corresponding author on reasonable request.
